# The Utilization of RNA Silencing Technology to Mitigate the Voriconazole Resistance of Aspergillus Flavus; Lipofectamine-Based Delivery

**DOI:** 10.15171/apb.2017.007

**Published:** 2017-04-13

**Authors:** Sanam Nami, Behzad Baradaran, Behzad Mansoori, Parivash Kordbacheh, Sasan Rezaie, Mehraban Falahati, Leila Mohamed Khosroshahi, Mahin Safara, Farideh Zaini

**Affiliations:** ^1^Department of Medical Mycology and Parasitology, School of Public Health, Tehran University of Medical Sciences, Tehran, Iran.; ^2^Immunology Research Center, Tabriz University of Medical Sciences, Tabriz, Iran.; ^3^Department of Medical Mycology and Parasitology, Faculty of Medicine, Iran University of Medical Sciences, Tehran, Iran.; ^4^Department of Immunology, Faculty of Medicine, Tabriz University of Medical Sciences, Tabriz, Iran.

**Keywords:** Cyp51a gene, MDR1 gene, RNA silencing, Voriconazole, Aspergillus flavus, Lipofectamine

## Abstract

***Purpose:*** Introducing the effect of RNAi in fungi to downregulate essential genes has made it a powerful tool to investigate gene function, with potential strategies for novel disease treatments. Thus, this study is an endeavor to delve into the silencing potentials of siRNA on cyp51A and MDR1 in voriconazole-resistant Aspergillus flavus as the target genes.

***Methods:*** In this study, we designed three cyp51A-specific siRNAs and three MDR1-specific siRNAs and after the co-transfection of siRNA into Aspergillus flavus, using lipofectamine, we investigated the effect of different siRNA concentrations (5, 15, 25, 50nM) on cyp51A and MDR1 expressions by qRT-PCR. Finally, the Minimum Inhibitory Concentrations (MICs) of voriconazole for isolates were determined by broth dilution method.

***Results:*** Cyp51A siRNA induced 9, 22, 33, 40-fold reductions in cyp51A mRNA expres­sion in a voriconazole-resistant strain following the treatment of the cells with concentrations of 5, 15, 25, 50nM siRNA, respectively. Identically, the same procedure was applied to MDR1, even though it induced 2, 3, 4, 10-fold reductions. The results demonstrated a MIC for voriconazole in the untreated group (4µg per ml), when compared to the group treated with cyp51A-specific siRNA and MDR1-specific siRNA, both at concentrations of 25 and 50nM, yielding 2µg per ml and 1µg per ml when 25 nM was applied and 2µg per ml and 0.5µg per ml when the concentration doubled to 50 nM.

***Conclusion:*** In this study, we suggested that siRNA-mediated specific inhibition of cyp51A and MDR1 genes play roles in voriconazole-resistant A.flavus strain and these could be apt target genes for inactivation. The current study promises a bright prospect for the treatment of invasive aspergillosis through the effective deployment of RNAi and gene therapy.

## Introduction

*Aspergillus* spores cause a broad-spectrum of diseases in humans, ranging from allergy-type illnesses to invasive infections depending on host immunity.^1,2^ Since the last decade, invasive aspergillosis (IA) is associated with significant morbidity and mortality in hematological malignancies, bone marrow transplant (BMT) recipients and patients suffering from AIDS and chronic granulomatous diseases.^3,4^ Of all the known *Aspergillus* species, approximately 80% of IA is caused by *Aspergillus* *fumigatus*; moreover, *Aspergillus flavus* is the second leading cause of IA in Western countries.^5,6^ In certain climate and geographical locations like the Middle East, Africa and Southeast Asia where arid climate dominates, the IA caused by *A. flavus* is more common than that caused by *A. fumigatus* since*A.flavus* has the ability to survive higher temperatures.^5,7^

The first line drug for the medical treatment and prophylaxis of IA is voriconazole.^8,9^ Voriconazole belongs to the subclass of triazoles which is a lanosterol 14 alpha-demethylase inhibitor.^10^ Lately, it has been witnessed that in the long run, Asian patients develop resistance to azole as a result of a lengthy exposure to this drug as a treatment.^11,12^ Preliminary investigation of voriconazole-resistant *A.flavus* has revealed amino acid residue substitution derived from mutations in the coding regions of cyp51A (Lanosterol 14 α-demethylase) genes or overexpression of this gene.^12,13^ It should be mentioned that voriconazole binds to cyp51A through its heme iron to the N-heterocycle nitrogen.^8,14^ Cyp51A is a cytochrome P450 enzyme that catalyzes ergosterol biosynthesis pathway.^14,15^ Fungal ergostrol is found in cell membranes and the inhibition of cyp51A in fungi results in membrane dysfunction and thus prevents fungal growth.^8,16^

Moreover, the comprehensive studies conducted on resistance to triazoles in *A.flavus* are indicative of the existence of a resistance mechanism other than mutations or overexpression of cyp51A gene; based on the evidence that roughly 40% of *A.flavus* strains do not depict any variation in their cyp51A gene.^17^ However, it is worth noting that the so-called strains resort to other resistance mechanisms, the most notable of which is multidrug resistance efflux pumps (MDR-EPs).^7^ The primary role of efflux proteins entails freeing the cells from amassed drug through energy consumption and consequently, preventing the concentration of drug from exceeding beyond the optimum level of growth inhibition.^17^ MDR-EPs is reckoned as one of the mechanisms that determines drug resistance in yeasts either by mutation or overexpression, two of the sub-categories of which are ATP-binding cassette (ABC) and major facilitator superfamily (MFS) which can have determining roles in fungal drug resistance. On the basis of recent studies, voriconazole-resistant *A.flavus* demonstrates a wide range of MDR1, 2, 4overexpression as compared with the wild-type strain.^17^

Furthermore, RNA interference (RNAi) is a post-transcriptional gene silencing (PTGS) phenomenon by which RNA molecules knock down essential genes responsible for vital as well as virulence factors.^18,19^ The RNAi machinery is mediated by short 21–25 nucleotide small-interfering RNAs (siRNA) which are the products of a double-stranded RNA (dsRNA) by the action of RNase III-like enzyme called dicer.^20-22^ These siRNAs will then be incorporated into a multi-protein siRNA complex known as the RNA-induced silencing complex (RISC).^23,24^ The RISC complex uses the incorporated siRNAs to target and degrade homologous messenger RNAs (mRNAs), leading to the silencing of the expression of corresponding endogenous genes.^25,26^ A homology-dependent gene silencing phenomenon in fungi, termed “quelling”, was first demonstrated in ascomycete *Neurospora crassa.*^22^ Recently, RNAi has been reported in several filamentous fungi like *Aspergillus fumigatus* and *Aspergillus nidulans.*^27,28^ Introducing the effect of RNAi in fungi to downregulate essential genes has made it a powerful tool to investigate gene function, with potential strategies for novel disease treatments.^22^ Thus, this study is an endeavor to delve into the silencing potentials of siRNA on cyp51A and MDR1 in voriconazole-resistant *A.flavus* as the target genes.

## Materials and Methods

### Fungal strains and antifungal susceptibility testing

In this study, a voriconazole-resistant *A.flavus* strain (Nr: 66041) and a voriconazole-susceptible strain (Nr: 65770) were used (These isolates were identified previously and were stored in the culture collection of the Medical Mycology Laboratory, School of Public Health, Tehran University of Medical Sciences, Tehran, Iran). Isolates were inoculated on potato dextrose agar (QUELAB, Canada) and incubated at 35°C to induce adequate sporulation. The conidia were collected and Inoculum suspensions were standardized by hemocytometer; and the final densities were adjusted to 0.4×10^4^-2.5×10^4^ colony-forming units per ml. The Minimum Inhibitory Concentrations (MICs) of voriconazole (*Sigma-*Aldrich, USA*)* for these isolates were determined by broth dilution method, as described in the Clinical and Laboratory Standards Institute (CLSI) document M38-A2.^29^ Candida krusei (ATCC 6258) and Candida parapsilosis (ATCC 22019) were used as quality control strains.^30^

### Spore germination and siRNA delivery

All strains were grown on potato dextrose agar and incubated at 35°C for 4 days to produce adequate conidia. Sterile double distilled water was added onto the surface of each plate, and the surface was scraped with a sterile loop to collect the conidia. The conidial suspension was centrifuged at 2000×/g for 5 min at 20°C and carefully washed with sterile water to remove the mycelial fragments. Thereafter, spores were cultured at a concentration of 1×10^6^ spores per ml on Czapek-dox broth medium (Sigma-Aldrich, USA) in 6-well plates and were incubated at 37°C for 6h. Totally, six 21-nucleotide siRNAs were designed and synthesised by Microsynth (the Swiss DNA company) to target the mRNA sequences of the cyp51A and MDR1 genes of *A. flavus*. These siRNAs were *labeled* as cyp51-1, cyp51-2, cyp51-3, MDR1-1, MDR1-2 and MDR1-3 Table 1. Purification by HPLC was carried out by the company and a negative control siRNA (NCsiRNA) was used to assess transfection efficiency. Transfections were performed using Lipofectamine™3000 reagent (Invitrogen Life Technologies, UK) according to manufacturer’s recommendations. Briefly, in a sterile microcentrifuge tubes, each of siRNAs was re-suspended in diethyl pyrocarbonate (DEPC)-treated water and then the final concentration of 5, 15, 25 and 50nM were prepared. Next, lipofectamine was mixed gently with different siRNA concentrations and incubated for 10 min at ambient temperature. The mixtures were inoculated to each well and were incubated for another 24h for the production of hyphae. After 24h, transfection efficiencies were determined by Cytation5 cell imaging multi-mode reader (BioTek, USA) using siGLO RNAi control (Dharmacon, USA).

**Table 1:** siRNA sequences

**siRNA Name**	**siRNA Sequence**
cyp51-A	Sequence Sense: 5'-GGA ACA UCC AGU CCU UAU UTT-3'
cyp51-2	Sequence Sense: 5'-UCA UCG UCC UAA AUC UGU UTT-3'
cyp51-3	Sequence Sense: 5'-AAG UAU GGC GAC AUC UUU ATT-3'
MDR1-1	Sequence Sense: 5'-GAA CAG AUG UCU CGU AUU ATT-3'
MDR1-2	Sequence Sense: 5'-UGC CGC AGC UGA AUU UAA ATT-3'
MDR1-3	Sequence Sense: 5'-CAA AGG CCG UUA UUA UGA ATT-3'

### RNA extraction and cDNA synthesis

Total RNA was extracted from conidia grown in potato dextrose agar medium after 4 days of incubation at 35°C without siRNA (untreated strains) and subsequently from fresh hyphae grown in Czapek-Dox broth medium after 24h of incubation with siRNA at 37°C (treated strains) using TRlzol lyzing reagent(Ambion life technologies). Very briefly, the hyphae were ground in a mortar with a pestle in liquid nitrogen and then TRlzol lyzing reagent (1ml) was added. The mixture was delivered to 1.5ml nuclease-free microtube and incubated for 5min at room temperature. Following the addition of chloroform (250µl), the microtube was shaken by hand for 15sec and incubated on ice for 10min. The suspension was centrifuged at 12500×g for 15min at 4°C. After centrifugation, the aqueous phase was transferred to a fresh tube. In order to precipitate RNA from the liquid phase, the ice-cold isopropanol (500µl) was added and samples were incubated on ice for 10min. Next, it was centrifuged at no more than 12500×g for 10min at 4°C. The supernatant was removed and the sediment was mixed with cold 75% ethanol (adding at least 1ml of 75% ethanol per 1ml of Trizol), then the sample was mixed by vortexing and centrifuged at 7800×g for 8min at 4°C (this phase was repeated one more time). The resultant supernatant was discarded and the precipitant dried on bench for 20min. In the end, the RNA was dissolved in 30µl diethyl pyrocarbonate (DEPC)-treated water and incubated for 10min at 55-60°C in digital dry bath. In order to determine RNA quantity and purity, the optical density of the solution was quantified by Nanodrop2000c spectrophotometer (Thermo fisher scientific). Thereafter, Complementary DNA (cDNA) was synthesized using cDNA synthesis kit (Sensiscript Reverse Transcription Kit, QIAGEN, Germany) from 1μg of total RNA. The reaction component encompassed 10× buffer RT (2 µl), dNTP mix (2 µl), oligo dT primer (2 µl), RNase inhibitor (1 µl), sensiscript reverse transcriptase (1 µl), extracted RNA (the volume of which was separately calculated and added to each individual sample according to the data given in the brochure of the kit.), having added RNase-free water, the volume reached to 20 µl and was incubated at 37°C for one hour. Eventually, the attained product, cDNA, was frozen at -20°C and kept for the successive working day.

### Quantitative Real-Time PCR (qRT-PCR)

Nucleotide sequences of cyp51A (NCBI accession numbers: XM_002375082) and MDR1 (NCBI accession numbers: XM_002382940) were obtained from the published gene sequence of *A. flavus* NRRL3357 (http://www.ncbi.nlm.nih.gov/pubmed/). Thereafter, gene-specific primers Table 2 were designed for cyp51A and MDR1 and tubulin was employed as a reference gene. Cyp51A and MDR1 mRNA levels were amplified by qRT-PCR instrument (light cycler 96, Roche) using SYBR Green Master Mix (AMPLIQON, Denmark). The PCR reaction conditions were as follows: 0.5μl of cDNA template, 0.25μl of each primer (forward and reverse), 5μl of master mix and 4.25μl of nuclease-free distilled water, totaling to the final volume of 10μl. The program for amplification was 95°C for 5min for initial denaturation, followed by 45cycles at 95°C for 10sec, 58°C for 35sec, and 70°C for 20sec. All qRT-PCR reactions were run in triplicate. The relative cyp51A and MDR1 mRNA expressions were analyzed by 2^-∆CT^ method. Finally, statistical analysis of the gene expression was carried out by one way and two way ANOVA statistical test by Graphpad prism version6 software.

Eventually, to confirm the success of target gene silencing by siRNA, MICs of voriconazole in treated strains were determined by broth dilution method, as previously described.

**Table 2:** Sequences of primers used in qRT-PCR analysis

**Primers Name**	**Sequence (5'-3')**	**PCR Product Size (bp)**
cyp51A-F	TGA GCC TGC AGT CAT GGA AG	211
cyp51A-R	GAC GTA AGG TGT GCC AGG AA	
MDR1-F	TTC CGC TTC TTC GTC TGC TT	166
MDR1-R	TCT TGC CAT CTT CCG ACC AC	
tubulin-F	AAC GCT TTG CAA CTC CTG AC	162
tubulin-R	AGT TGT TAC CAG CAC CGG AC	

## Results and Discussion

### Confirmation of siRNA delivery into Aspergillus flavus

**Figure 1:**
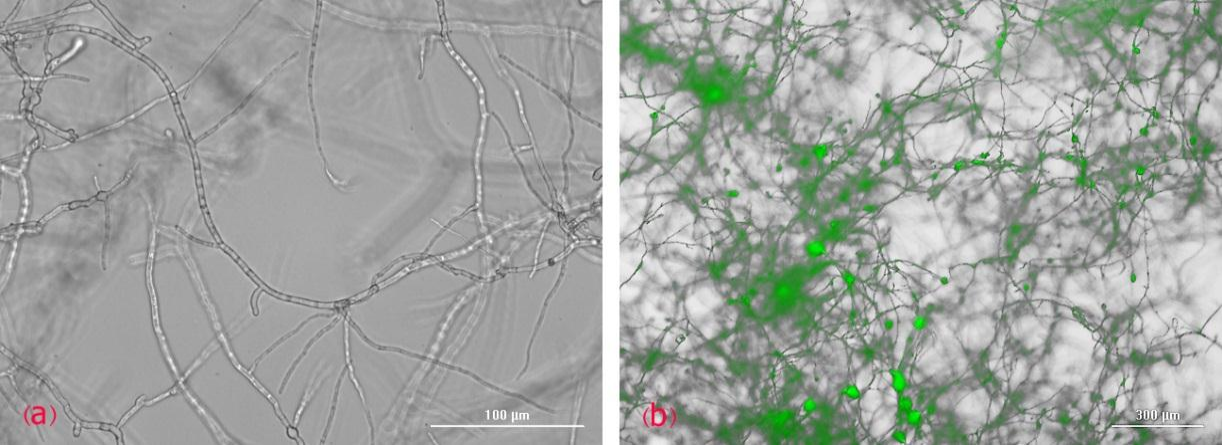
(a) Untreated *A.flavus* image by Cytation5 cell imaging multi-mode reader (b) Treated *A.flavus* with specific labeled siRNA (50nM) image by Cytation5 cell imaging multi-mode reader.

Having used Cytation5 cell imaging multi-mode reader, we verified that the siRNA had been successfully delivered into the mycelia treated with labelled siRNA (siGLO RNAi control) on Czapek-Dox broth media. The images were taken 24h after the various concentrations (5, 15, 25, 50nM) of siRNA were added to broth media Figure 1.

### Effect of siRNA concentration on cyp51A and MDR1 expressions

**Figure 2:**
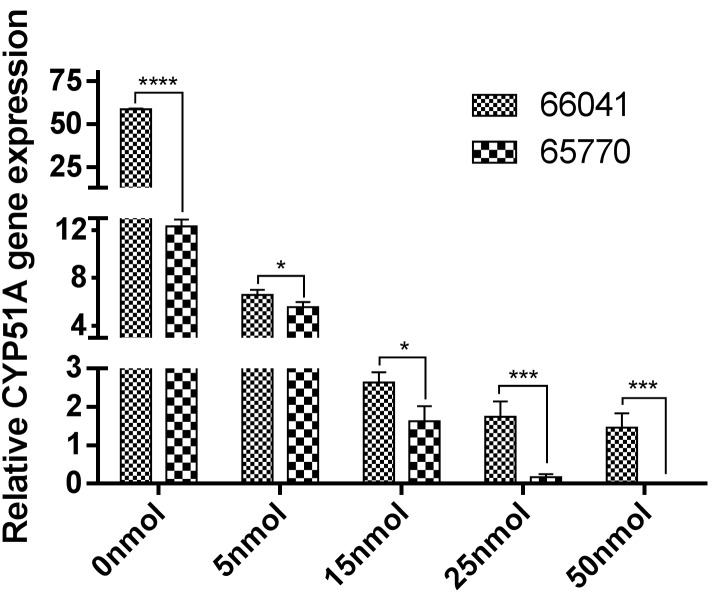
Effect of 5, 15, 25, 50nM concentration of cyp51A siRNA on gene expression in a voriconazole-resistant *A.flavus* (66041) and a voriconazole-susceptible *A.flavus* (65770).0nmol: untreated strain (positive control).

**Figure 3:**
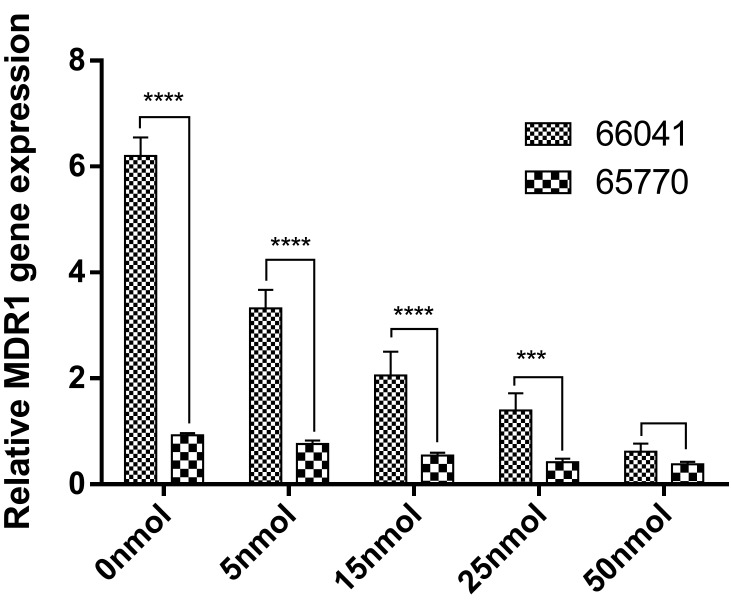
Effect of 5, 15, 25, 50nM concentration of MDR1 siRNA on gene expression in a voriconazole-resistant *A.flavus* (66041) and a voriconazole-susceptible *A.flavus* (65770).0nmol: untreated strain (positive control).

Cyp51A and MDR1 mRNA expressions were quantified by qRT-PCR in a voriconazole-resistant *A.flavus* strain and a voriconazole-susceptible strain treated by the specific siRNA and positive control (untreated group) and compared with a negative control (unrelated siRNA) after 24h. The relative quantification of cyp51A and MDR1 gene expressions were normalized to the housekeeping gene tubulin. Several studies have indicated that point mutations and overexpression in cyp51A gene could be responsible for modulating the susceptibility of *Aspergillus spp*. to azole drugs.^12,31^ Our results demonstrated a remarkable upregulation of cyp51A gene (5folds) (P<0.0001) in our voriconazole-resistant strain as opposed to susceptible strain. Howard et al. demonstrated that upregulation in cyp51A gene is associated with azole resistance in *A.fumigatus*,^32^ but Liu et al. indicated that the expression levels of cyp51A, cyp51B and cyp51C genes were not associated with voriconale-resistance in *A. flavus*,^8^ whose results were in conflict with our findings. In light of the previously conducted studies which had indicated that the overexpression of MDR1, 2, 4, atrF, MFS-EPs stemmed from resistance to voriconazole in *A.flavus* strains as compared to the wild type groups,^17^ our study also confirmed the upregulation of MDR1 gene (7folds) (P<0.0001) in voriconazole-resistant strain as opposed to susceptible strain. This finding was also validated by Natesan et al. who highlighted the necessity of further studies such as knockdown or gene cloning to better explicate the role of MDR-EPs in resistance to voriconazole *A.flavus* strains.^17^ With regard to the findings of Abdel-Hadi et al., the efficient siRNA transfection was remarkably higher in the presence of lipofectamin and this reagent enhanced the uptake of siRNA in a medium.^20^ So, we designed synthetic siRNAs to target the cyp51A and MDR1 genes of *A. flavus* and mix with lipofectamin reagent. Our results were indicative of the fact that all the designed siRNAs yielded excellent levels of silencing of the cyp51A and MDR1 genes and the use of lipofectamin reagent increased the efficacy of transfection. This study showed that cyp51A siRNA induced 9, 22, 33, 40-fold reductions in the cyp51A mRNA expression in a voriconazole-resistant strain following the treatment of the cells with concentrations of 5, 15, 25, 50nM siRNA, respectively Figure 2. The summary of resultant mean and standard deviation (SD) values for the treated-resistant strain after applying 5, 15, 25, 50nM concentration of cyp51A specific siRNA were 6.58±0.43, 2.63±0.26, 1.75±0.39, 1.46±0.37, respectively, as compared to the positive control. Identically, the same procedure was applied to MDR1, even though it induced 2, 3, 4, 10-fold reductions Figure 3, and accordingly, resulted in various mean and SD at 3.32±0.35, 2.06±0.45, 1.39±0.32, 0.62±0.15, respectively. The same results for the treated-susceptible strain were 5.58±0.42, 1.63±0.39, 0.17±0.08, 0.02±0.01, respectively. Eslami et al. recommended that the expression of the target gene (sidB gene) decreased significantly, using 25nM of siRNA, showing the most transfection efficiency at this concentration.^21^ Based on the obtained data, in this study, we proved that the downregulation of cyp51A and MDR1 genes’ expressions occurred at concentrations ranging from 5 to 50nm of gene-specific siRNA. Moreover, our results confirmed that there was less knockdown at 5nM and greater knockdown at 50nM, compared with the cyp51A and MDR1 genes expression levels of the untreated control. Treatment with the negative control siRNA had no significant effect on cyp51A and MDR1 mRNA expressions and downregulation was observed in all siRNAs, indicating that the results were not caused by off-target effects.

### Antifungal susceptibility testing of Aspergillus flavus

Susceptibility testing was performed for all untreated and treated voriconazole-resistant and susceptible isolates, as previously described. The results demonstrated a MIC for voriconazole in the untreated group (4µg per ml), when compared to the group treated with cyp51A-specific siRNA and MDR1-specific siRNA both at concentration of 25 and 50nM, yielding 2µg per ml and 1µg per ml when 25 nM was applied and 2µg per ml and 0.5µg per ml when the concentration doubled to 50 nM Table 3. The current study shed light on the drastic effect of siRNA in reducing resistance in treated group with cyp51A-specific siRNA not to mention the induction of susceptibility in strain when treated with MDR1-specific siRNA. The susceptibility to voriconazole in the untreated susceptible strain was nowhere near that of the treated group, although the overexpression of the treated susceptible group was negligible as opposed to the resistant group, which lends support to the scanty overexpression of these two genes in the susceptible group. Mellado et al. showed that targeted disruption of *cyp51A* in itraconazole-susceptible and resistant *A. fumigatus* strains decreased MICs from 2-fold to 40-fold,^33^ which lends support to our findings. Given the fact that each cell division diminishes the concentration of siRNA and since roughly 4-5 days were required after transfecting siRNA for obtaining MIC test results, a marginal change was observed which can be better enhanced by ameliorating the MIC procedure.

**Table 3:** In Vitro Susceptibility of Laboratory Voriconazole-Resistant Aspergillus flavus Isolates

**Tessted strains**	**MIC, μg/mL of VCZ**
A.flavus (resistant strain, untreated)	4
A.flavus (resistant strain, treated; cyp51A-25nM)	2
A.flavus (resistant strain, treated; cyp51A-50nM)	2
A.flavus (resistant strain, treated; MDR1-25nM)	1
A.flavus (resistant strain, treated; MDR1-50nM)	0.5

Abbreviations: MIC, minimum inhibitory concentration; VCZ, voriconazole Susceptible ≤ 1 μg/mL, Intermediate: 2μg/mL, Resistant: ≥ 4 μg/mL

## Conclusion

In this study, we suggested that siRNA-mediated specific inhibition of cyp51A and MDR1 genes can play roles in voriconazole-resistant *A.flavus* strain and these could be apt target genes for inactivation. However, we recommend evaluating the whole range of cyp subtype genes (including cyp51A, cyp51B and cyp51C) and various MDR-EPs and performing experiments on the effect of siRNA on human cells in the future studies. The current study promises a bright prospect for the treatment of invasive aspergillosis through the effective deployment of RNAi and gene therapy.

## Acknowledgments

This study was financially supported by Tehran University of Medical Sciences (grant number: 28288).

## Ethical Issues

None to be declared.

## Conflict of Interest

Authors declare no conflict of interest in this study.
